# Institutional Notes and News

**Published:** 1911-01-21

**Authors:** 


### Institutional Notes and News.

**General Hospital, Hereford.**

The Herefordshire Education Committee received from the General Hospital the following resolution, which was passed at a meeting of the honorary medical staff: "That the Association should oppose the reference of school children, found upon medical inspection, to public medical charities for treatment, whether or not accompanied by payments or subsidies." An interesting discussion followed as to the reasons which had led to the framing of this resolution, in which Mr. Bulmer, who is a member of the hospital Board of Management, said: "The view of the medical profession is if all these children, who are an increasing number, are to be treated gratis at the hospital, the State is throwing what ought to be a public medical service upon the medical men at the hospital without any remuneration, and depriving medical men of possible fees. They contend that the State has no right to expect medical men who have to earn their living to treat these cases gratis through a medical charity. The Board of Management, after a long consideration of the matter, thought it courteous to let the Education Committee have notice that they could not continue to treat these cases after June 30." Another gentleman, Mr. Corner, pointed out that the Education Committee did not send the children to the hospital: it had them examined merely, and if the parents chose to get subscribers' tickets, surely the hospital authorities should not refuse them. "If they do, then their subscriptions will soon go down." The discussion therefore presents two interesting points—namely, that the honorary medical staff at Hereford is not to be mollified by the payment of a subsidy to the hospital, but wants, apparently, a State-paid Medical Service to deal with school children, and secondly, that there is an opinion that the hospital has not the right to refuse admission to school children if they apply with subscribers' letters in their pockets. It will be interesting to watch the developments of this deadlock, for which, we hope, a solution will be found before June 30.

**The King and Portsmouth Hospital.**

We congratulate the Portsmouth, Portsea, and Gosport Hospital on having received His Majesty's sanction to use the prefix Royal in its name and title. Our readers will remember that the Home Secretary in October last drew the attention of the Hospital's Committee to the fact that it had no present right to the style of Royal. Much local feeling was aroused by this reminder on the ground that the hospital, having called itself Royal for sixty years, had a prescriptive right to be called and recognised as Royal by other people. But, as we pointed out in our issue of October 15, "a charitable institution, like a corporation or society, is only granted the right to prefix the adjective Royal to its title by special permission from the Crown." This has now been gained, and places the hospital in the strong position of an institution the work of which has been recognised and approved by Royalty. It is obvious that the value of the prefix is destroyed if it is the symbol of no qualification, and to remind the public, as some of the hospital's supporters were tactless enough to do, that the Royal Marine Hotel of Little Puddlington had not been brought to book for a similar use of an unauthorised adjective, was to put the hospital on a level with a useful but undignified place of refreshment in a way that it is the first wish of every one to avoid. The fact, however, that the hospital *could* be compared, strictly speaking, to a hostelry was the final justification of the Home Secretary's action; and now that the Royal permission has been granted we believe that the hospital's friends who were first aggrieved will be the first also to admit the improved status and position of their institution.

**Middlesex Hospital.**

Perhaps it is not as widely known as it ought to be that the Middlesex Hospital, while open at all hours to accident and emergency cases, generally dispenses with the formality of subscribers' letters, which are not always obtainable by the more independent and deserving poor. Its growth has been astonishing: in the troubled year 1745 it occupied two houses still to be seen in Windmill Street, but ten years later it took possession of its present building, then in the midst of fields. In its first centenary it boasted 250 beds, and, though surrounded by houses, has contrived to preserve its gardens, which are still its great attraction. So long ago as 1792 it became endowed with funds for a special cancer ward, the disease then, as now, offering an urgent and seemingly not insoluble problem. The patients suffering from cancer are now domiciled in separate buildings, where incurable cases are tended till death releases them. These, with the Medical School and Convalescent Home, supplement the greatly enlarged original foundation, and form a hospital fully up to the most modern standard. A concert to aid the hospital funds was arranged by Miss Alys Bateman with the late Prince Francis, who was a supporter of good music, and had personally attended her concerts and recitals. It is now, under sadder circumstances, to be given by desire of Prince Alexander and with the approval of their Majesties, and assumes a grander and more impressive character, the interest céntring round Verdi's "Requiem." To bring out the full pathos and dramatic surprises of this famous work 500 instruments and voices are required, and these are provided by the full strength of the Brighton Choir and Festival Orchestra, under Mr. Joseph Sainton. The choir has not yet been heard in London. The soprano parts, taken by Alys Bateman, are most arduous. Verdi's "Requiem," composed soon after "Aida," was performed for the first time on May 15, 1876, when the composer himself conducted and was tremendously fêted. It is now, however, nine years since it has been heard in London, and this fact alone should fill the Queen's Hall. It will be preceded by Beethoven's beautiful overture "Leonora III.," and fittingly concludes with Wagner's triumphal "Huldigungs-marsch." It is hoped that, as a mark of respect, as many of the late Prince's friends as are able will attend. The following have joined the committee of the concert: Lord Pembroke, Lord Kilmorey, Lord Cheylesmore, the Hon. G. W. Spencer-Lyttelton, Lieutenant-Colonel the Hon. C. G. Gathorne-Hardy, and Lord Duncannon.

**The Queen's Hospital for Children.**

We are glad to learn from Lord Shaftesbury, who presided at a special meeting of Governors on Monday last, that the hope we expressed on November 19 for the success of the dinner in aid of the Queen's Hospital for Children, Hackney Road, has been fulfilled. It appears £1,870 were raised at it, and that in his lordship's words, the dinner "was the means of obtaining a large number of new subscribers." Since then, moreover, a handsome cheque for £250 has been received from Mr. Robert Nevison. The special meeting, however, had other business before it besides that of taking note of past successes: it had to consider the present position of the hospital, and to decide how that might be improved. It is true that since November, the month in which the dinner was held, the hospital's indebtedness has been reduced from £8,500 to £6,000. But this is only the first step in a financial campaign. Sir Walter Johnson therefore moved that a special Appeal Committee should be formed, and that the efforts begun during 1910, which have produced already a modicum of success well worth their trouble, should be redoubled, so that in 1912 the hospital may start afresh debt free. The particular claims of this institution on the support of the public have been urged in The Hospital frequently during the past twelve months. It is necessary to recapitulate here only one point of the first importance: that the hospital serves a gigantic tract of North-east London, which is probably the least known, has been the least written up, and is the least popular of any slum district in the capital. We all know of the East End, but who knows of the North-east End, a district as large as, and much less explored than, that of its more notorious sister. Whitechapel in the past has had its poets, and, as an inevitable consequence, has now its art museum, and one of the few fine thoroughfares in London. When will Hackney inspire a sonnet, or a novel? When will it be known that some successful man of letters spent his spare hours as a boy reading Blake in the Bethnal Green Museum? To create this atmosphere in the public mind should be one of the first works of the Appeal Committee, the appointment of which we have just noted. For it is this atmosphere which excites the sympathy that is expressed in special subscriptions.

